# Induction Effects of *Bacteroides fragilis* Derived Outer Membrane Vesicles on *Toll Like Receptor 2, Toll Like Receptor 4* Genes Expression and Cytokines Concentration in Human Intestinal Epithelial Cells

**DOI:** 10.22074/cellj.2019.5750

**Published:** 2018-11-18

**Authors:** Sara Ahmadi Badi, Shohreh Khatami, Shiva Irani, Seyed Davar Siadat

**Affiliations:** 1 Department of Biology, Science and Research Branch, Islamic Azad University, Tehran, Iran; 2 Department of Biochemistry, Pasteur Institute of Iran, Tehran, Iran; 3 Department of Mycobacteriology and Pulmonary Research, Pasteur Institute of Iran, Tehran, Iran; 4 Microbiology Research Centre, Pasteur Institute of Iran, Tehran, Iran

**Keywords:** *Bacteroides fragilis*, Gut Microbiota, Membrane Vesicles, Toll Like Receptors

## Abstract

**Objective:**

Gastrointestinal (GI) tract, like other mucosal surface, is colonized with a microbial population known as gut microbiota. Outer membrane vesicles (OMVs) which are produced by gram negative bacteria could be sensed by Toll like receptors (TLRs). The interaction between gut microbiota and TLRs affects homeostasis and immune responses. In this study, we evaluated *TLR2, TLR4* genes expression and cytokines concentration in Caco-2 cell line treated with *Bacteroides fragilis (B. fragilis)* and its OMVs.

**Materials and Methods:**

In this experimental study, OMVs were extracted using sequential centrifugation and their physicochemical properties were evaluated as part of quality control assessment. Caco-2 cells were treated with *B. fragilis* and its OMVs (180 and 350 µg/ml). Quantitative reverse transcriptase-polymerase chain reaction (qRT-PCR) was performed to assess *TLR2* and *TLR4* mRNA expression levels. Pro-inflammatory (IFNᵧ) and anti-inflammatory (IL- 4 and IL-10) cytokines were evaluated by ELISA.

**Results:**

* B. fragilis* significantly decreased *TLR2* and slightly increased *TLR4* mRNA levels in Caco-2 cell line. The *TLR2* mRNA level was slightly increased at 180 and 350 µg/ml of OMVs. Conversely, the *TLR4* mRNA level was decreased at 180 µg/ml of OMVs, while it was significantly increased at 350 µg/ml of OMVs. Furthermore, *B. fragilis* and its OMVs significantly increased and decreased IFNᵧ concentration, respectively. Anti-inflammatory cytokines were increased by *B. fragilis* and its OMVs.

**Conclusion:**

*B. fragilis* and its OMVs have pivotal role in the cross talk between gut microbiota and the host especially in the modulation of the immune system. Based on the last studies on immunomodulatory effect of *B. fragilis* derived OMVs on immune cells and our results, we postulate that *B. fragilis* derived OMVs could be possible candidates for the reduction of immune responses.

## Introduction

Gastrointestinal (GI) tract is colonized by a variety, complex and dynamic microbial community referring as gut microbiota. This microbial community also consists of bacteria, fungi, archea, protozoa and viruses (1). Gut microbiota constantly interacts with the epithelium of GI tract. This putative cross talk has potential role in both host functions (locally and systemically) and establishment of gut microbiota pattern. Thus, host functions and gut microbiota pattern regulate health and diseases status (2).

Gut microbiota is considered as a reservoir for immune system stimulatory molecules due to the presence of immunogenic compounds such as lipopolysaccharides (LPS), peptidoglycans (PG) and extracellular vesicles (EVs) (3, 4). These bacterial components are encountered in the gut barrier (epithelial layer) as the first line of gut innate immunity. The gut barrier is also composed of intestinal epithelial cells, mucus layer that is produced by goblet cells, innate and adaptive immune factors (i.e. antimicrobial peptides and immunoglobulins, mainly including IgA). Indeed, the gut barrier shapes gut microbiota and its interaction to host (5, 6). Moreover, the gut barrier functions are under the control of pattern recognition receptors (PRRs) including toll like receptors (TLRs), nucleotide binding domain leucine rich repeat containing receptors (NLRs), retinoic acid inducible gene like receptors (RLRs), C-type lectin receptors (CLRs) and absent in melanoma 2 (AIM2)-like receptors (ALRs) (7, 8). PRRs sense pathogen associated molecular patterns (PAMPs) or damage associated molecular patterns (DAMPs), trigger various signaling cascades and induce different responses (9). Various cell types including immune and intestinal epithelial cells express TLRs that are belonged to type I transmembrane receptors (10). The expression patterns of TLRs among GI epithelial cell are different and the interaction between gut microbiota and TLRs affects local and systemic immunity (8). Disrupted homeostasis, considered as dysbiosis, results from the imbalance between gut microbiota and immune responses. It is considered as a turning point to induce many disorders including metabolic syndrome (11). This condition which is characterized by impaired permeability of gut barrier, known as leaky gut syndrome, causes a great activation of TLRs in intestinal epithelial cells (IEPCs) (12). Consequently, increased cytokines and chemokines trigger low grade inflammation. Increased inflammatory cytokines disrupt insulin signaling cascade and may cause insulin resistance (IR), ultimately promoting metabolic syndrome and obesity (13). 

*Bacteroides spp.* such as *B. fragilis* have significant roles in gut microbiota-host interactions, especially on metabolic and immune system (14). Similarly, Bacteroides spp. derived outer membrane vesicles (OMVs) are key players in gut microbiota host interactions (15). OMVs are nanosized and spherical vesicles which could affect metabolic and immune system since they contain bacterial components including LPS, outer membrane proteins, phospholipids, periplasmic components, DNA, RNA, hydrolytic enzymes and signaling molecules (16). 

*B. fragilis* also secretes capsular polysaccharide A(PSA) containing OMVs. These OMVs interact with dendritic cells (DCs) through *TLR2* signaling pathway, resulting in CD4+ and regulatory T- cells (Tregs) induction. The latter one is crucial for host immune tolerance towards commensal intestinal bacteria. Therefore, *B. fragilis* derived OMVs contribute to maintain gut microbiota homeostasis (17, 18). In this regard, we evaluated and compared the effects of *B. fragilis* and its OMVs on *TLR2, TLR4* genes expression and cytokines concentration on Caco-2 cell line as a IEPCs model.

## Materials and Methods

### Bacterial growth conditions

In this experimental study, *B. fragilis* ATCC 23745 was grown on blood agar plates containing 5% sheep blood or brain heart infusion (BHI) broth supplemented with 5 µg/ ml hemin (Sigma-Aldrich, USA) and 1 µg/ml menadione (Sigma-Aldrich, USA), while they were incubated at 37°C, in 80% N_2_, 10% CO_2_ and 10% H_2_ atmosphere (19).

### Outer membrane vesicles extraction

OMVs were isolated as described previously (20). Briefly, after an overnight cultivation, the medium was centrifuged at 6000 g, 4°C. The pellets were washed twice with phosphate buffer solution (PBS) and re-suspended in 9% sodium chloride solution. Then the suspension was centrifuged for 1 hour at 6000 g, 4°C. OMVs were extracted through sequential centrifugation for 90 minutes at 20000 g, 4°C using Tris-ethylene diamine tetra acetic acid (EDTA)-sodium deoxycholate (Sigma-Aldrich, USA) buffers. Finally, OMVs were stored at -20°C (20).

### Scanning electron microscopy

The OMVs were fixed in PBS containing 2.5% glutaraldehyde and 2% paraformaldehyde. Following PBS washing, the samples were air-dried and coated with gold by sputter coater (KYKY Technology, China (using physical vapor deposition method. The prepared samples were examined by SEM (KYKY Technology, China) (21). 

### Cell culture and treatment

The human epithelial cell line, IBRC C10094 Caco-2, was obtained from Iranian Biological Resource Center. The cells were grown in Dulbecco’s modified eagle medium (DMEM/high glucose; Gibco™, USA), supplemented with 10% fetal bovine serum (FBS, Gibco™, USA) and 1% penicillin/streptomycin (Gibco™, USA) and incubated at 37°C in a 5% CO_2_ atmosphere (22). The cells were treated with *B. fragilis* and OMVs (180 and 350 µg/ ml) and incubated overnight. 

### RNA isolation and cDNA synthesis

Total RNA was isolated using RNX-Plus (CinnaGen, Iran). RNA quantity and quality were respectively evaluated by NanoDrop 2000 (Thermo Fisher Scientific, USA) and gel electrophoresis. cDNAs were synthesized by RevertAid first strand cDNA synthesis kit (Thermo Scientific, USA) according to manufacturers’ instructions.

### Quantitative reverse transcriptase polymerase chain reaction analysis

Quantitative reverse transcriptase polymerase chain reaction (qRT-PCR) was performed using LightCycler® 96 SW 1.1 instrument (Roche, Germany). Each reaction mixture was composed of SYBR Premix Ex Taq II (Takara, China), specific primers (Table 1) and DNA template. GAPDH was used as housekeeping gene. The amplification program was consisted of 1 cycle at 95°C for 60 seconds, followed by 40 cycles of denaturation at 95°C for 5 seconds, annealing at 55°C for 30 seconds and extension at 72°C for 30 seconds. 

**Table 1:** List of primers for quantitative reverse transcriptase-polymerase chain reaction (qRT-PCR) analysis


Gene	Prime sequence (5ˊ-3ˊ)

*GAPDH*	F: GGAGCGAGATCCCTCCAAAAT
	R: GGCTGTTGTCATACTTCTCATGG
*TLR2*	F: TTATCCAGCACACGAATACACAG
	R: AGGCATCTGGTAGAGTCATCAA
*TLR4*	F: AGACCTGTCCCTGAACCCTAT
	R: CGATGGACTTCTAAACCAGCCA


### Cytokines concentration assay

Following overnight incubation of Caco-2 cells with *B. fragilis* and its OMVs, the supernatants were collected and stored at -20°C. The IFNγ, IL-10 and IL-4 concentrations were measured using enzyme-linked immunosorbent assay (ELISA) kit (Human cytokine ELISAPRO kit, MABTECH, Swedish biotech, Sweden), according to manufacturer’s instructions.

### Statistical analyses

Data were analyzed by independent sample t test and one- way ANOVA using GraphPad Prism software (GraphPad Software, Inc., San Diego, CA). All results demonstrate as mean ± standard deviation (SD). In all experiments, P<0.05 was considered statistically significant.

## Results

### Properties of *B. fragilis* derived outer membrane vesicles

**Fig.1:**
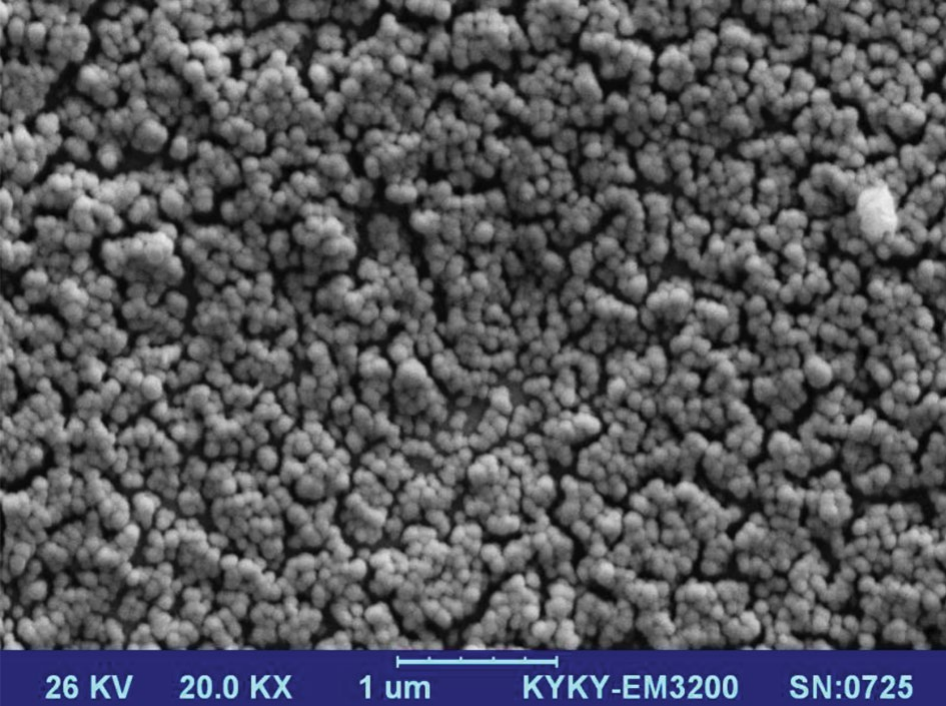
*B. fragilis* produces outer membrane vesicles (OMVs) with a mean dimention of 85.7 ± 15.3 nm: scanning electron microscopy of *B. fragilis* derived-OMVs (magnification: ×20K).

*B. fragilis* produced OMVs in BHI broth. The morphology and size of OMVs were examined by SEM. Diameter of spherical shaped OMVs was in the range of 30-110 nm (Fig .1). Mean dimension of OMVs was 85.7 ± 15.3 nm. 

### Effect of B. fragilis and outer membrane vesicles on TLR gene expressions

**Fig.2:**
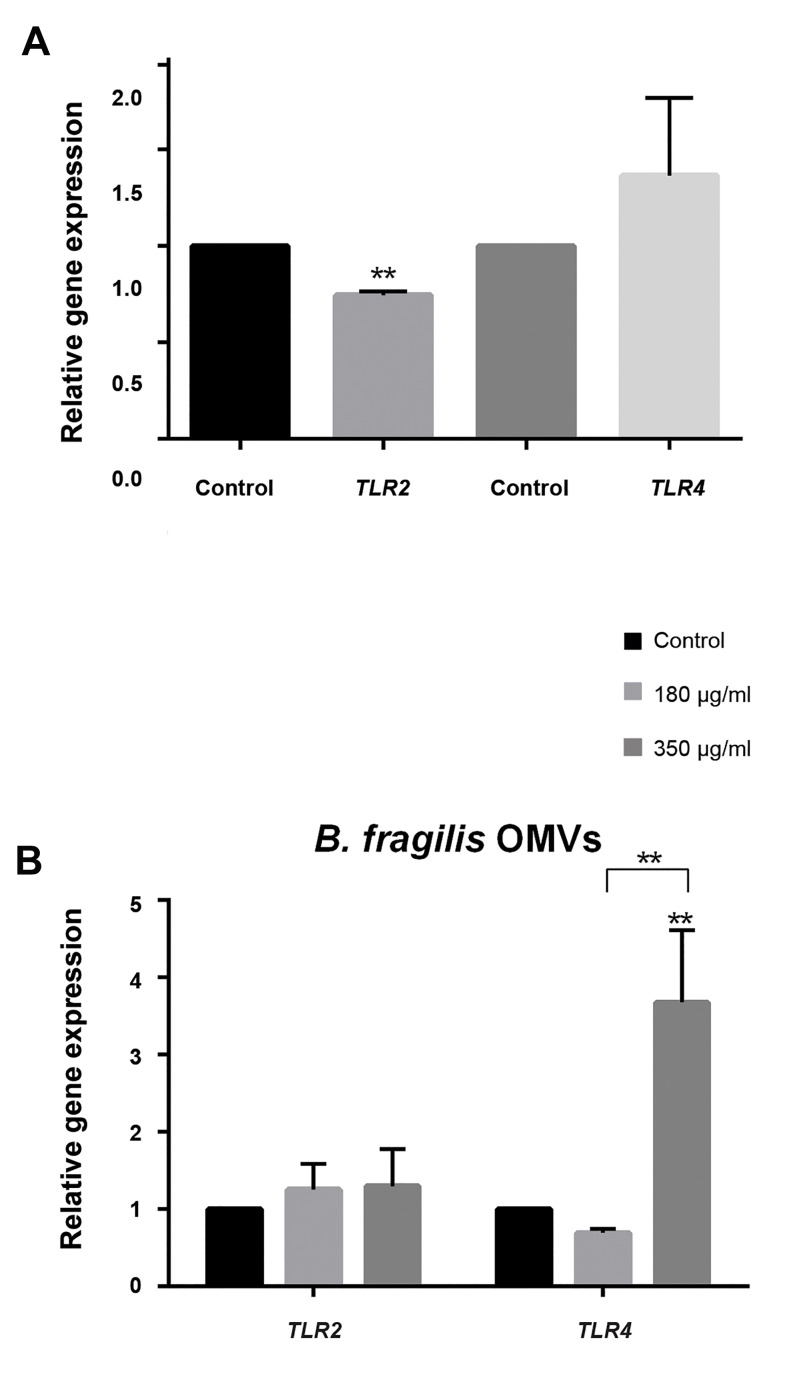
Quantitative reverse transcriptase-polymerase chain reaction (qRT-PCR) analyzes of *B. fragilis* and its outer membrane vesicles (OMVs) on TLR genes expressions. A. The cells were initially deprived of serum and then treated with either *B. fragilis* or phosphate buffer solution (PBS) overnight and B. In the same condition, the other group cells were treated with either *B. fragilis* derived OMVs (350 and 180 µg/ml) or sucrose, overnight. Values of triplicate experiments are demonstrated as mean ± SD. Significant results are presented as ** based on P<0.01.

Human intestinal epithelial cell line Caco-2 was used to study the effects of *B. fragilis* and its OMVs on *TLR2* and *TLR4* gene expressions using qRT-PCR. *B. fragilis* significantly decreased *TLR2* gene expression. *TLR4* gene expression was slightly increased by this bacterium (Fig .2A). The cells were treated with *B. fragilis* derived OMVs in two concentrations, 180 and 350 µg/ml. The mRNA levels of *TLR2* were slightly increased in both of OMVs concentrations. Interestingly, *TLR4* gene expression was decreased and significantly increased at 180 and 350 µg/ml of OMVs, respectively (Fig .2B).

### Effect of B. fragilis and outer membrane vesicles on cytokines concentration

**Fig.3:**
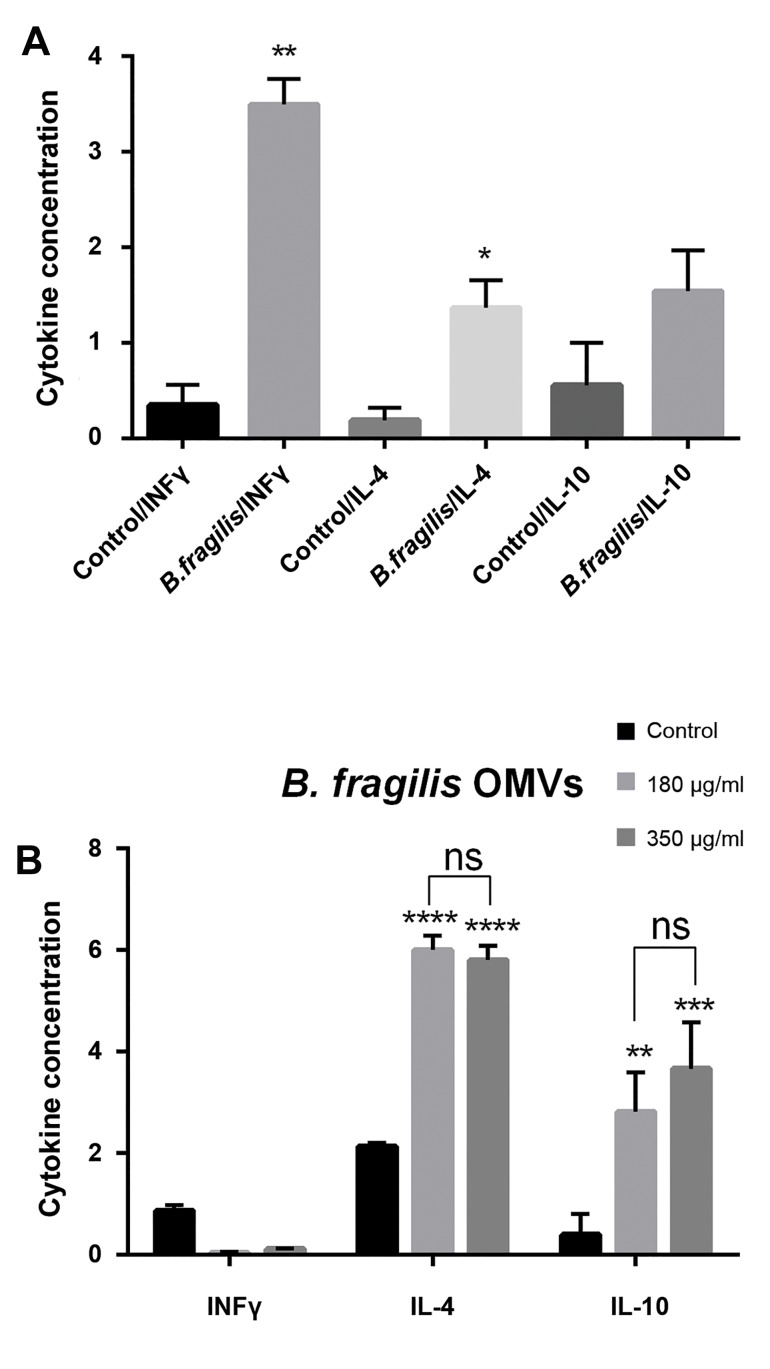
ELISA analyzes of *B. fragilis* and its outer membrane vesicles (OMVs) on cytokines concentration. A. Cells were initially deprived of serum and then treated with either *B. fragilis* or phosphate buffer solution (PBS), overnight and B. In the same condition, the other group cells were treated with either *B. fragilis* derived OMVs (350 and 180 µg/ml) or sucros, for overnight. Values of triplicate experiments are demonstrated as mean ± SD. Significant results are presented as *, **, ***, **** based on P<0.05, P<0.01, P<0.001, and P<0.0001.

After overnight stimulation of Caco-2 cells by *B. fragilis* and its OMVs, the concentration of pro- inflammatory (IFNγ) and anti-inflammatory (IL-4 and IL-10) cytokines were measured by ELISA. *B. fragilis* significantly elevated IFNγ concentration (Fig .3A). Interestingly, IFNγ concentration was decreased by 180 and 350 µg/ml of OMVs (Fig .3B). *B. fragilis* was able to increase IL-4 and IL-10 concentrations (Fig .3A). In addition, the related OMVs of this bacterium (180 and 350 µg/ml) significantly enhanced IL-4 and IL-10 concentrations (Fig .3B). 

## Discussion

The epithelial layer of GI tract is continuously exposed to huge amount of immunogenic stimulatory molecules, derived from gut microbiota, nutrient and pathogenic microorganisms (3). IEPCs are the interface between gut microbiota and immune system via lamina propria cells. The potential of IEPCs to modulate immunity depends on PRRs gene expression (5). Additionally, the gut microbiota has immunomodulation potential in host. In this regard, *B. fragilis* and its OMVs affect gut microbiota-host interactions (15). Therefore, we aimed to study in more details the effects of *B. fragilis* and its OMVs on TLR genes expression and cytokines concentration in Caco-2 cell line as a human IEPCs model. 

It has been found that TLRs play a crucial role in immune responses and *B. fragilis* influences homeostasis and immunity (14). In other words, *B. fragilis* activate CD4+ T cells responses through *TLR2* signaling in DCs. *B. fragilis* has anti-inflammatory effects through mediation of Th1/Th2 balanced ratio, as well as CD4+ T cells differentiation into Tregs and Th17 limited responses (17). Moreover, TLRs signaling in GI epithelium triggers the cross talk between gut microbiota and the host, locally and systemically (6). TLRs signaling is involved in proliferation, differentiation of IEPCs alongside with induction of pro- and anti-inflammatory cytokines responses. As IEPCs are located in frontline of gut environment, their TLRs signaling has critical role in immune tolerance to gut microbiota and defense against pathogens (8). Expression patterns and induction mode of TLRs are different throughout GI epithelium. IEPCs have relatively low expression of *TLR2* and *TLR4*, which are the main receptors for gram positive and negative bacterial MAMPs (9). In this regard, Furrie et al. (23) reported that *B. fragilis* does not change the *TLR1-4* expression levels in Caco-2 cell line. In our study, although *B. fragilis* significantly decreased *TLR2* , but increased *TLR4* gene expression. Perhaps, differences in bacterial quantity and incubation time could justify this discrepancy.

As mentioned above, gut microbiota could intervene with cytokines secretion. For instance, *B. fragilis* has immune-modulatory effect through induction of IL10 and reduction of IL-17 production during intestinal inflammation (17). Bahrami et al. (24) studied the influence of intestinal commensal bacteria (i.e. *B. fragilis*) on pro- and anti-inflammatory cytokine productions. Their data showed that *B. fragilis* did not affect cytokine concentration. However, we noticed that IFNγ, IL-4 and IL-10 concentrations were increased after corresponding treatment.

It has been demonstrated that *B. fragilis* releasing OMVs is an influential factor for mediation of immune responses. Since *B. fragilis* apparently does not have well established secretory system, immunogenic components (PSA) delivery is facilitated through OMVs production. Shen et al. have shown that *B. fragilis* has protective role against intestinal inflammatory disease in animal model via OMVs production. Indeed, *B. fragilis* OMVs induce Treg development and IL-10 production thorough *TLR2* signaling in DCs (17, 18). We believe that this is the first study reporting the effects of *B. fragilis*-derived OMVs on *TLR2* and *TLR4* genes expression, as well as the concentration of IFNγ, IL-10 and IL-4 on Caco-2 cell line. Taken together, our results depicted that *TLR2* mRNA levels were not altered by *B. fragilis* derived OMVs. However, these vesicles significantly changed *TLR4* gene expression. Interestingly, *B. fragilis* derived OMVs had stimulatory effect on anti-inflammatory cytokines (IL-4 and IL-10) while it decreased IFNγ concentration as a pro-inflammatory cytokine.

## Conclusion

Based on immunomodulatory effects of *B. fragilis* derived OMVs on immune system and our current findings, we suggest that these OMVs may have a substantial role in the improvement of the inflammatory responses and it may have yet no recognized and understudied function in the inter-kingdom modulation of host genes. 
